# Chronic thromboembolic pulmonary hypertension secondary to a vascular malformation: case report diagnosis by lung subtraction iodine mapping

**DOI:** 10.3389/fmed.2023.1206116

**Published:** 2023-06-15

**Authors:** Aly Fawzy, Sebastian Mafeld, George Oreopoulos, Marc de Perrot, Micheal C. McInnis

**Affiliations:** ^1^Temerty Faculty of Medicine, University of Toronto, Toronto, ON, Canada; ^2^Division of Vascular and Interventional Radiology, Department of Medical Imaging, University of Toronto, Toronto, ON, Canada; ^3^Toronto General Hospital, University Medical Imaging Toronto, Toronto, ON, Canada; ^4^Division of Vascular Surgery, Toronto General Hospital, University Health Network, University of Toronto, Toronto, ON, Canada; ^5^Division of Thoracic Surgery, Toronto General Hospital, University Health Network, University of Toronto, Toronto, ON, Canada; ^6^Division of Cardiothoracic Imaging, Department of Medical Imaging, University of Toronto, Toronto, ON, Canada

**Keywords:** balloon pulmonary angioplasty (BPA), chronic thromboembolic pulmonary hypertension (CTEPH), vascular malformation, computed tomography, case report

## Abstract

Chronic thromboembolic pulmonary hypertension (CTEPH) is a challenging diagnosis that can occur even in the absence of a prior thrombotic event. The main screening test is ventilation-perfusion (VQ) scintigraphy. The gold standard treatment for CTEPH is pulmonary endarterectomy (PEA), however, balloon pulmonary angioplasty (BPA) is an emerging treatment, especially for CTEPH at the segmental level. We report on a case of a patient with segmental CTEPH diagnosed by lung subtraction iodine mapping (LSIM) in the context of a chest wall vascular malformation. CTEPH was treated with BPA and by embolization and ligation of their vascular malformation.

## Introduction

Chronic thromboembolic pulmonary hypertension (CTEPH) is World Health Organization (WHO) Group 4 pulmonary hypertension characterized by chronic thromboembolism (1). The prevalence of CTEPH is estimated to be between 2 and 5 per 100,000, however, the disease is considered underdiagnosed (2). CTEPH presents with dyspnea at rest or with exertion and is a challenging diagnosis because of the insidious onset and non-specific symptoms (1). In fact, CTEPH can occur even in the absence of a reported thromboembolism. While chronic pulmonary embolism in the main and lobar pulmonary arteries may be easy to detect, disease restricted to the segmental and subsegmental vessels can be extremely subtle. Pulmonary endarterectomy (PEA) is the gold standard for treating CTEPH and experienced centers see excellent results even in patients with disease in the segmental vasculature (1–3). Recently, balloon pulmonary angioplasty (BPA) has been an emerging treatment for CTEPH, particularly for segmental artery lesions that may be challenging to access surgically or in patients where surgical risks are high (1, 2, 4). We present a case of subsegmental CTEPH in the context of a chest wall vascular malformation that was treated with BPA.

## Case presentation

**Figure 1:**
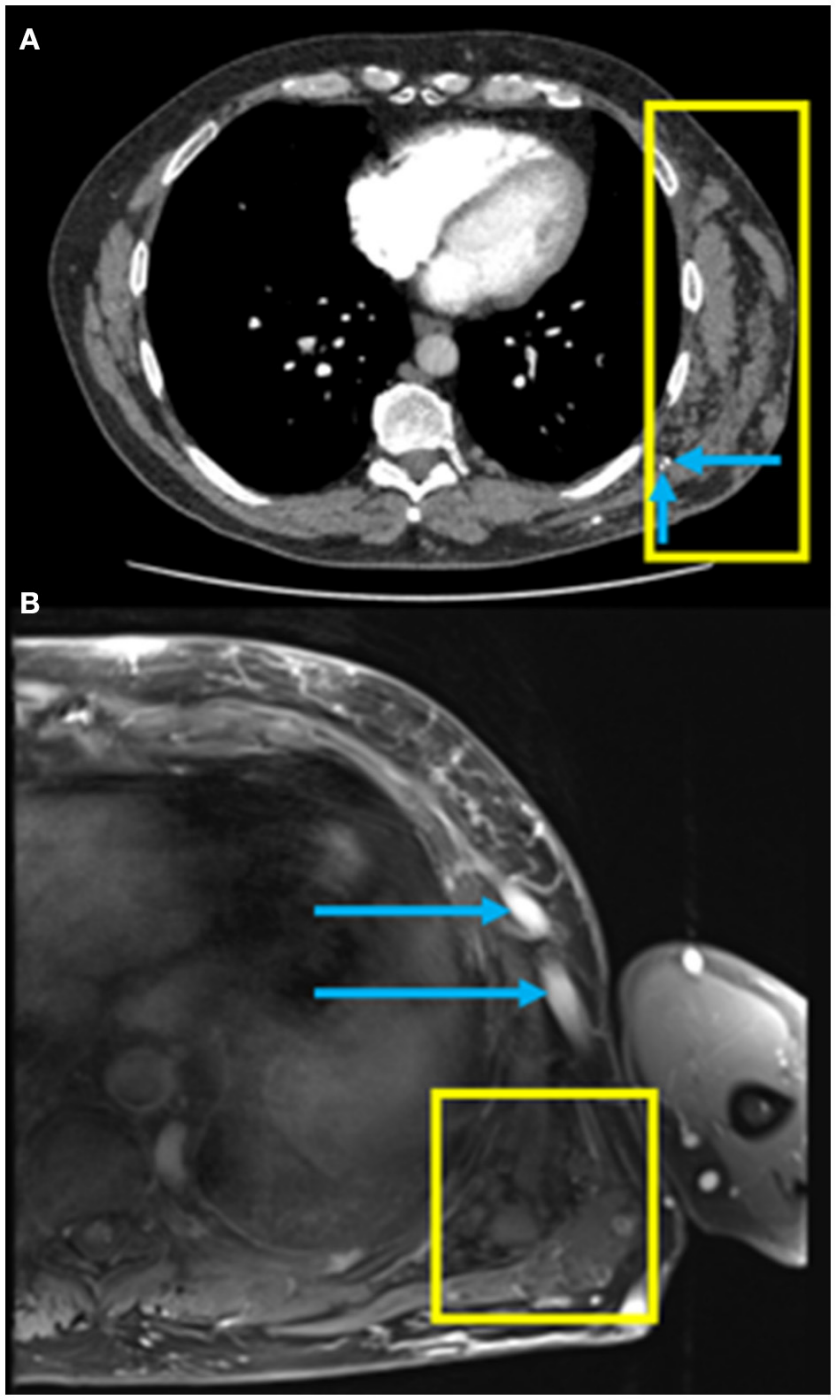
**(A)** Axial computed tomography pulmonary angiography (CTPA) of the chest in soft tissue windows demonstrating a chest wall low flow vascular malformation (yellow box) with the presence of phleboliths (blue arrows); **(B)** Axial fat saturated T1-weighted MRI post gadolinium image of the left lateral chest wall demonstrating a low flow vascular malformation with clustered vessels centered under the left latissimus dorsi muscle (yellow box) and large draining veins in the subcutaneous tissues (blue arrows).

A 57-year-old male presented with increasing shortness of breath at rest and with exertion over the past 2–3 years. He used to be able to go up 5–6 flights of stairs and now he is limited to 1–2 flights of stairs, being New York Heart Association (NYHA) functional class II at the time of presentation. He was known to have a vascular malformation in the left chest wall (Figure 1). There was no other significant past medical history and no history of a venous thrombotic event. The patient was a non-smoker and reported no history of lung disease. There was no history of prior surgery, no relevant family history and the patient took no medications prior to initial assessment.

**Figure 2:**
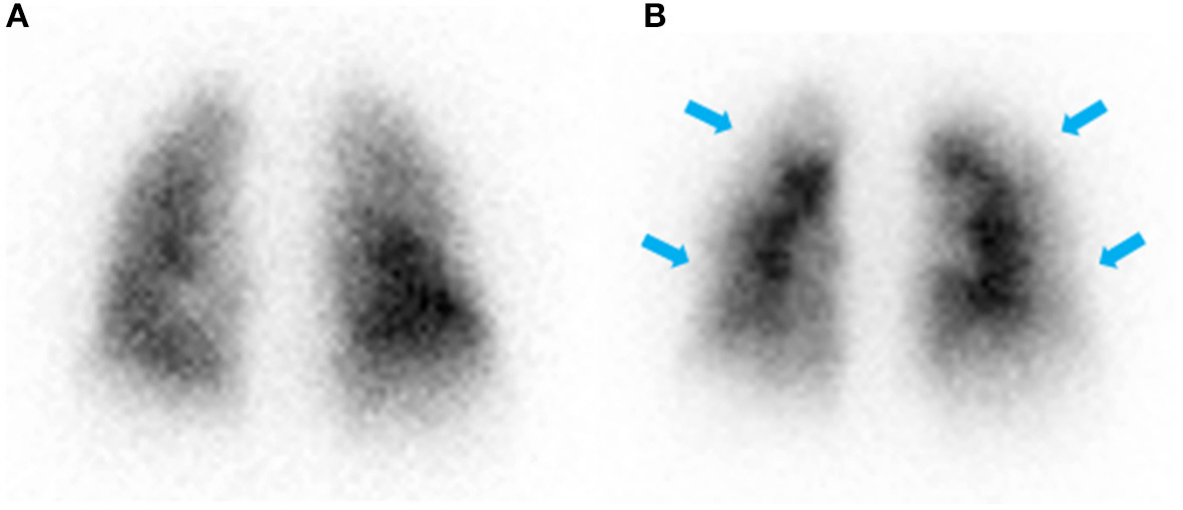
Posterior view of ventilation **(A)** and perfusion **(B)** scintigraphy showing normal ventilation and a paucity of perfusion in the periphery of the lung (blue arrows) with no defined segmental or lobar lesions.

The patient appeared well at rest. Echocardiogram revealed a moderately enlarged right ventricle (RV) with mildly impaired RV systolic function. Right heart catheterization confirmed pulmonary hypertension (PH) with a mean pulmonary artery pressure (mPAP) of 50 mmHg, a mean pulmonary artery wedge pressure (PAWP) of 13 mmHg and cardiac output of 5.7 L/min. Ventilation-perfusion (VQ) scintigraphy that demonstrated a paucity of perfusion in the peripheral lung (Figure 2). The patient subsequently underwent computed tomography pulmonary angiography (CTPA) to further investigate the PH and VQ mismatch which showed borderline dilation of the main pulmonary artery measuring 28 mm, and no chronic pulmonary embolism in the proximal right and left pulmonary arteries.

**Figure 3:**
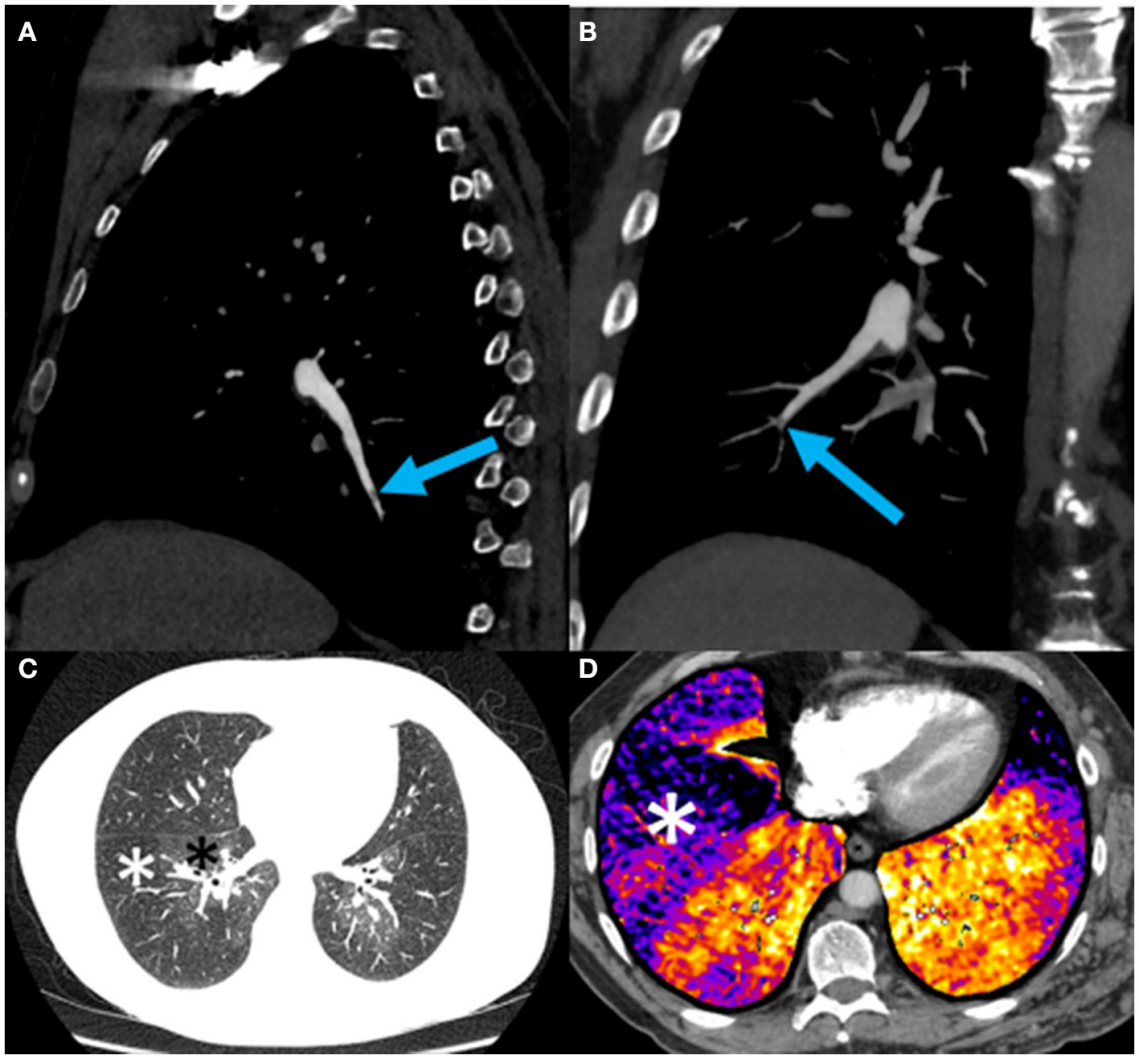
**(A)** Sagittal oblique reformat of a chest CTPA showing a subtle web (blue arrow) in the distal right lower lobe posterior basal segment; **(B)** Coronal oblique maximum intensity projection view of a chest CTPA demonstrating a subtle web-like lesion (blue arrow) in the anterior basal right lower lobe pulmonary artery at the subsegmental level. This is the lesion subsequently treated with balloon pulmonary angioplasty (BPA) (see Figure 4); **(C)** Axial CTPA of the chest in lung windows at the level of the left atrium demonstrating striking mosaic lung attenuation with geographic regions of hypoperfused lung (white asterisks) and normally perfused lung (black asterisks); **(D)** Axial CTPA lung subtraction iodine mapping (LSIM) at the lung bases demonstrating areas of low iodine concentration in the periphery of the lung detonated by black-purple coloration (white asterisk) and corresponding to perfusion defects on VQ scintigraphy (Figure 2). Red-orange coloration indicates normal lung perfusion.

On CTPA reformats, the patient was found to have a subtle web in the distal right lower lobe posterior basal segment (Figure 3A) and a subtle web-like lesion in the anterior basal right lower lobe pulmonary artery at the subsegmental level (Figure 3B). Lung subtraction iodine mapping (LSIM) highlighted perfusion defects in areas of pulmonary embolism (Figures 3C, D). LSIM involves performing a non-contrast CT followed by a conventional CTPA, then digitally subtracting the non-contrast CT from the CTPA to generate an iodine map. With high confidence in the presence of chronic pulmonary emboli, the patient was thus diagnosed with CTEPH.

**Figure 4:**
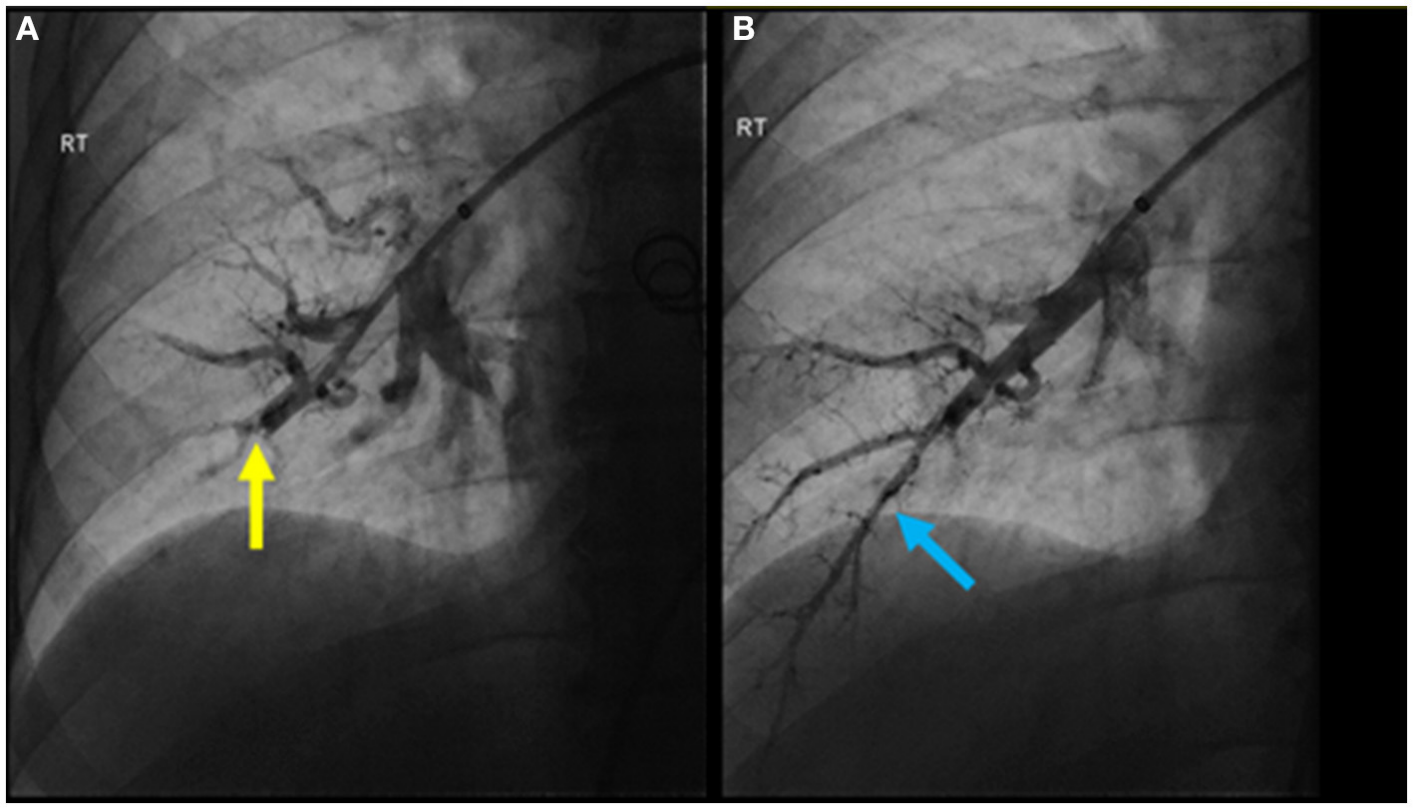
**(A)** Pulmonary angiogram pre-BPA of the anterior basal right lower lobe segmental pulmonary artery demonstrating significant impairment in flow due to a subtle web lesion (yellow arrow); **(B)** post-BPA pulmonary angiogram showing significant improvement in flow (blue arrow).

The patient was treated medically with apixaban, riociguat, and macitentan. After multidisciplinary discussion, the chest wall vascular malformation was hypothesized to be the most likely source of the chronic pulmonary emboli. Thus, around 1 month after the diagnosis was confirmed, the communication of the chest wall vascular malformation with the systemic veins was interrupted using interventional radiology directed embolization and surgical ligation. Four months later, the patient underwent sequential BPA sessions to treat their pulmonary thromboembolic lesions. In the initial session, access was achieved through the right common femoral vein. A 6-French vascular sheath was advanced followed by a pigtail catheter and a hydrophilic wire to gain access to the right pulmonary artery. A 0.014 in × 185 cm PT2 microwire was used to selectively access the right lower lobe anterior basal segmental branch, with significant impairment in flow due to a subtle web lesion (Figure 4A). Two balloons, measuring 2 and 3 mm, were inflated to dilate the artery and disrupt the web. Significant improvement in flow was observed post-BPA (Figure 4B).

There were six BPA sessions over the course of 15 months treating a total of twenty lesions. The procedures were well tolerated with no significant adverse events. The patient reported substantial symptomatic improvement being able to perform activities without shortness of breath (NYHA class I). Indeed, the patient's 6-min walk test improved from 475 m walked at baseline to 570 m walked at follow-up and there was reduction of mPAP from 50 to 38 mmHg. Follow-up echocardiography also demonstrated improvement with a mildly enlarged RV (previously moderately enlarged) and normal global RV systolic function (previously mildly impaired).

## Discussion

We present a case of subsegmental CTEPH diagnosed in the absence of a distinct thromboembolic event in a patient with a chest wall vascular malformation, treated with BPA. CTEPH is a challenging diagnosis to make due to the non-specific symptoms (dyspnea, fatigue, and chest pain) and should be suspected in any patient with a history of pulmonary embolism and PH. To make the diagnosis of CTEPH, a patient must have mismatched perfusion defects on VQ scintigraphy, a mPAP > 20 mmHg at rest with PAWP ≤ 15 mmHg, a pulmonary vascular resistance of >2 WU and imaging evidence of chronic PE after at least 3 months of effective anticoagulation therapy (1, 2, 5).

While chronic pulmonary embolism in the main and lobar pulmonary arteries may be easy to detect, disease restricted to the segmental and subsegmental vessels can be extremely subtle. Patients with this more distal disease are more likely than those with proximal disease to be women, to have a prior splenectomy, be on direct oral anticoagulants (DOACs) and preoperative PH-targeted medical therapy (1, 3). Pulmonary emboli manifest on CT as webs, occlusion or eccentric thickening (1, 6). Addition of a CT perfusion technique such as LSIM or dual energy CT (DECT) improves detection of these subtle findings. LSIM is accurate in diagnosing segmental/subsegmental CTEPH, similar to DECT, with a higher sensitivity and specificity than CTPA alone (1, 6, 7). An advantage of the LSIM technique, in contrast to DECT, is the ability to perform this test on any modern scanner provided that the software is available. DECT requires specific CT scanner hardware that is not as widely available. Although LSIM involves performing two scans, the radiation dose is similar to DECT (8). Both techniques have potential drawbacks including misregistration artifacts in LSIM and potential limitations in temporal resolution for DECT. Our patient had clear perfusion defects as seen on their VQ and LSIM. Moreover, they showed subtle but definite evidence of chronic PE in small vessels on CTPA and conventional angiography.

Our patient presented with no prior history to a thrombotic event but had a chest wall vascular malformation. This was thought to be the source of chronic pulmonary emboli wherein small thrombi form in a low flow environment and subsequently embolize to the lung. There are multiple case reports of CTEPH developing in patients with vascular malformations, such as Klippel-Trenaunay-Weber syndrome and mediastinal venous malformations (9–12). While vascular malformations appear to be a risk factor for CTEPH, there is no specific evidence for screening in this population (13). Our patient's vascular malformation was interrupted using interventional radiology directed embolization and surgical ligation in order to prevent future incidences of pulmonary emboli.

BPA has been an emerging treatment for CTEPH, specifically for patients with where pulmonary endarterectomy may be challenging, such as in those with multiple comorbidities and disease in the subsegmental vessels. BPA has excellent short-term outcomes with hemodynamic, functional, and quality-of-life improvements as seen in this case (4). Our patient underwent BPA for treatment of their subsegmental disease and has been maintained on medical therapy since then. Notably, there is a paucity of long-term data on the use of BPA in CTEPH. Recruitment for the International BPA Registry (ClinicalTrials.gov: NCT03245268) is now complete and the outcome data from the Registry may help guide further management in these complex patients. Although a substantial symptomatic improvement was achieved in our patient, continued close monitoring will be required considering the limitations of BPA as a minimally invasive technique compared to pulmonary endarterectomy.

## Conclusion

Segmental and subsegmental CTEPH is a challenging diagnosis to make, especially in the absence of a thromboembolic event, but should be considered in a patient with a vascular malformation and dyspnea. LSIM may improve the detection of chronic PE and BPA should be considered in select cases of CTEPH. Long term outcome data is required to further inform the management strategy in this group of patients.

## Data availability statement

The original contributions presented in the study are included in the article/supplementary material, further inquiries can be directed to the corresponding author.

## Ethics statement

Written informed consent was obtained from the individual(s) for the publication of any potentially identifiable images or data included in this article.

## Author contributions

MM: conceptualization and supervision. AF: writing—original draft preparation. MM, SM, GO, and MP: writing—review and editing. All authors have read and agreed to the published version of the manuscript.
